# Optic Nerve Injury in a Patient with Chronic Allergic Conjunctivitis

**DOI:** 10.1155/2014/928486

**Published:** 2014-09-15

**Authors:** Ribhi Hazin, Christopher J. Elia, Maria Putruss, Amanda Bazzi

**Affiliations:** ^1^Faculty of Arts and Sciences, Harvard University, 29 Garden Street, Suite No. 214, Cambridge, MA 02138, USA; ^2^Neurosurgery Department, Arrowhead Regional Medical Center, 400 N Pepper Avenue, Colton, CA 92324, USA; ^3^Saint John Providence Hospital, 16001 W 9 Mile Road, Southfield, MI 48075, USA; ^4^University of Michigan, Ann Arbor, MI 48109, USA

## Abstract

Manipulation of the optic nerve can lead to irreversible vision changes. We present a patient with a past medical history of skin allergy and allergic conjunctivitis (AC) who presented with insidious unexplained unilateral vision loss. Physical exam revealed significant blepharospasm, mild lid edema, bulbar conjunctival hyperemia, afferent pupillary defect, and slight papillary hypertrophy. Slit lamp examination demonstrated superior and inferior conjunctival scarring as well as superior corneal scarring but no signs of external trauma or neurological damage were noted. Conjunctival cultures and cytologic evaluation demonstrated significant eosinophilic infiltration. Subsequent ophthalmoscopic examination revealed optic nerve atrophy. Upon further questioning, the patient admitted to vigorous itching of the affected eye for many months. Given the presenting symptoms, history, and negative ophthalmological workup, it was determined that the optic nerve atrophy was likely secondary to digital pressure from vigorous itching. Although AC can be a significant source of decreased vision via corneal ulceration, no reported cases have ever described AC-induced vision loss of this degree from vigorous itching and chronic pressure leading to optic nerve damage. Despite being self-limiting in nature, allergic conjunctivitis should be properly managed as extreme cases can result in mechanical compression of the optic nerve and compromise vision.

## 1. Case

A 24-year-old male member of an indigenous nomadic tribe in the West African nation of Mali was seen at the emergency department complaining of extreme itching, tearing, and irritation in both eyes. The patient denied any significant past medical history. He did, however, describe visiting a tribal healer on a regular basis for “itchy skin” over his chest and upper extremities since childhood. He usually itched his skin rigorously until the appearance of blood in the affected area. He did experience some soothing of the itching when using topical ointments from the herbal doctor but admitted chronic noncompliance to the herbal therapy.

The patient's main complaint upon admission was “constant itching” in both eyes accompanied by excessive tearing for almost a year. The left eye was described as being “far worse” in terms of the intensity of the pain, photophobia, and itching. He felt the itching “deep on the bone of the left eye and nose” and would attempt to relieve this itching sensation regularly by inserting his index finger into the left infraorbital rim until his “eyeball felt like it was going to pop out” in an attempt to relieve the itching. He did this because the itching was felt “all the way back into his eye and nose.” The left eye had subluxed several times by his report necessitating digital pressure to reposit. His failure to visit the city hospital for so long stemmed from his inability to pay for the services and medication.

On admission, the patient's visual acuity in the right eye was 20/30 and 20/600 in the left eye. Visual field testing revealed severe constriction of the visual field on the left eye. Initial examination revealed no abnormal eversion of the upper lid but was significant for lid edema, bulbar conjunctival hyperemia, and large conjunctival papillae on the superior tarsus. Swinging flashlight test revealed an afferent pupillary defect of the left eye. Slit lamp examination revealed inferior and superior conjunctival scarring but no signs of external trauma or neurological damage were noted. Blepharospasm was significant on the left eye but minimal in the right. Two distinct superior corneal scars were noted, one that was healed and the other in the acute phase. Diffuse accumulations of gelatinous, white viscous material were also noted along the superior limbus. Conjunctival cultures and cytologic evaluation were negative for polymorphonuclear leukocytes but demonstrated significant eosinophilic infiltration. Subsequent ophthalmoscopic examination revealed optic nerve atrophy. The history and findings suggested optic nerve damage secondary to digital pressure with repeated eye subluxation during vigorous itching leading to optic nerve damage. A diagnosis of self-inflicted blindness secondary to optic nerve damage was made. Oral Benadryl and naphazoline hydrochloride drops were prescribed and he was discharged two days after admission with resolution of itching symptoms.

## 2. Discussion

Although rare, mechanical compression of the optic nerve via digital pressure or self-inflicted subluxation of the orbit can cause optic nerve damage and result in blindness [1]. Mechanical compression of the optic nerve most commonly arises in the setting of psychosis or unstable psychiatric disposition but, as seen in our patient, can also be part of the presentation of untreated allergic conjunctivitis (AC) [1–5]. Most of the reported cases of self-inflicted blindness via optic nerve damage attribute the patient's psychiatric disposition to injurious behavior or habits that result in loss of vision [1–5]. The itching, rubbing, or poking of the optic nerve described in the reported cases occurred in the absence of underlying infectious or anatomic pathology with the exception of one case which arose in the setting of normal tension glaucoma [1–7]. Although injurious behavior such as self-mutilation was the predominant cause of self-inflicted blindness in these patients, only one case attributed itching or rubbing of the eye to the blindness [6, 7]. In our patient, months of vigorous rubbing of the left eye yielded significant loss of vision. Blindness in the setting of repetitive rubbing of the eyes is thought to arise secondary to abnormal stretching of the optic nerve [2, 6, 7]. Chronic rubbing of the eyes is associated with optic neuropathy and may eventually cause blindness; however, there is often improvement of symptoms once rubbing desists [2, 6, 7]. Blepharospasm has also been linked to optic neuropathy and has been recognized as a possible source of blindness [8, 9]. The role of blepharospasm in contributing to blindness in our patient remains uncertain [9].

Given the rarity of optic neuropathy in young adults, self-inflicted injuries to the optic nerve should be considered as part of the differential diagnosis in patients presenting with unexplained blindness [2]. Prior to making a diagnosis, the physician needs to obtain a detailed history from the patient identifying any possible source of chronic ocular injury to aid in the diagnosis. Self-inflicted ocular injury must be ruled out prior to arriving at a diagnosis. This is especially true in mentally handicapped children in whom self-inflicted ocular injuries can result in self-mutilation resulting in autoenucleation, retinal detachment, and other forms of self-inflicted visual loss [1–3, 8]. Identifying individuals at risk of such behavior is critical to minimizing of the deleterious outcomes of such behavior [2, 4, 8]. Nonetheless, unexplained visual loss in pediatric and adolescent populations can result from ocular perforation, retinal detachment, cataracts, optic nerve avulsion, or other anatomic malformations as possible etiologies [1, 4]. These possibilities should be ruled out in pediatric patients [1].

Self-inflicted blindness has been reported in the literature as most commonly stemming from a patient's psychiatric instability, a deliberately injurious habit, or combination of both [1, 3–5, 8]. Individuals with obsessive-compulsive disorder, schizophrenia, bipolar disease, and Münchausen's disease have all been described in the literature as having habits which lead to self-inflicted blindness [1, 3–5, 8]. For instance, patients experienced blindness after inserting objects deep within their orbits, sustaining gunshot wounds to the temple, or repetitively subluxing the eye in an effort to get the attention of their parents [2–5]. Only one of the cases of self-inflicted blindness present in the literature described an underlying nonpsychiatric medical condition as being responsible for the repetitive rubbing and itching [6, 7]. In our case, the patient vehemently denied past psychiatric history or even a history of anxiety. Furthermore, our patient's presentation marks the first reported case of optic nerve damage secondary repeated trauma (stretching/pressure). Of the cases in the literature, the only other one describing a purely medical cause of the self-inflicted damage was one in which a patient with normal tension glaucoma caused damage to his optic nerve following weeks of recurrent eye rubbing [6, 7].

Diagnosing a patient suspected of self-inflicted optic nerve damage requires extensive ocular, neurologic, and systemic evaluation that should include cerebral and orbital magnetic resonance imaging studies to monitor the severity of optic nerve atrophy [2]. Our ability to conduct such studies was limited in our patient considering the remote location of the village hospital in which the case was seen. Notwithstanding, ophthalmoscopic examination revealed signs of optic nerve atrophy.

In patients suspected of self-inflicted blindness, thorough psychiatric evaluation may be warranted given the association reported in the literature between social and behavior problems and such deleterious outcomes [1–5]. Ruling out mental health disorders may assist in minimizing future injurious habits [2, 8]. This is critical since diagnosing psychiatric disorders can be difficult to make in an ophthalmology department [6–8]. Notwithstanding, while seeking to treat underlying symptoms, the ophthalmologist and ancillary staff are encouraged to remain sympathetic and supportive toward patients deemed self-destructive [1, 6–8]. Ensuring long-term psychiatric follow-up may be beneficial in improving future prognosis as well as minimizing future recurrence of symptoms [1, 3, 8, 9]. In patients like the one described in this case who have no underlying psychiatric instability, thorough history and complete ophthalmologic examination are recommended to steer the diagnosis in an appropriate direction.
